# Arrhythmogenic Cardiomyopathy Is a Multicellular Disease Affecting Cardiac and Bone Marrow Mesenchymal Stromal Cells

**DOI:** 10.3390/jcm10091871

**Published:** 2021-04-26

**Authors:** Arianna Scalco, Cristina Liboni, Roberta Angioni, Anna Di Bona, Mattia Albiero, Nicole Bertoldi, Gian Paolo Fadini, Gaetano Thiene, Stephen P. Chelko, Cristina Basso, Antonella Viola, Marco Mongillo, Tania Zaglia

**Affiliations:** 1 Department of Cardiac, Thoracic, Vascular Sciences and Public Health, University of Padova, 35128 Padova, Italy; arianna.scalco@gmail.com (A.S.); dibonanna@gmail.com (A.D.B.); gaetano.thiene@unipd.it (G.T.); cristina.basso@unipd.it (C.B.); 2 Veneto Institute of Molecular Medicine, 35129 Padova, Italy; mattia.albiero@gmail.com (M.A.); gianpaolo.fadini@unipd.it (G.P.F.); 3 Department of Biomedical Sciences, University of Padova, 35131 Padova, Italy; cristina.liboni@unipd.it (C.L.); roberta.angioni@unipd.it (R.A.); bertoldinicole@gmail.com (N.B.); antonella.viola@unipd.it (A.V.); 4 Fondazione Istituto di Ricerca Pediatrica-Città della Speranza, 35127 Padova, Italy; 5 Department of Medicine, University of Padova, 35128 Padova, Italy; 6 Department of Biomedical Sciences, Florida State University College of Medicine, Tallahassee, FL 32306, USA; stephen.chelko@med.fsu.edu; 7 Institute of Neuroscience, Italian National Research Council (CNR), 35121 Padova, Italy

**Keywords:** arrhythmogenic cardiomyopathy, mesenchymal stromal cells, bone marrow, myocardial remodeling, desmoglein-2

## Abstract

Arrhythmogenic cardiomyopathy (AC) is a familial cardiac disorder at high risk of arrhythmic sudden death in the young and athletes. AC is hallmarked by myocardial replacement with fibro-fatty tissue, favoring life-threatening cardiac arrhythmias and contractile dysfunction. The AC pathogenesis is unclear, and the disease urgently needs mechanism-driven therapies. Current AC research is mainly focused on ‘desmosome-carrying’ cardiomyocytes, but desmosomal proteins are also expressed by non-myocyte cells, which also harbor AC variants, including mesenchymal stromal cells (MSCs). Consistently, cardiac-MSCs contribute to adipose tissue in human AC hearts. We thus approached AC as a multicellular disorder, hypothesizing that it also affects extra-cardiac bone marrow (BM)-MSCs. Our results show changes in the desmosomal protein profile of both cardiac- and BM- MSCs, from desmoglein-2 (*Dsg2*)-mutant mice, accompanied with profound alterations in cytoskeletal organization, which are directly caused by AC-linked DSG2 downregulation. In addition, AC BM-MSCs display increased proliferation rate, both in vitro and in vivo, and, by using the principle of the competition homing assay, we demonstrated that mutant circulating BM-MSCs have increased propensity to migrate to the AC heart. Taken altogether, our results indicate that cardiac- and BM- MSCs are additional cell types affected in *Dsg2*-linked AC, warranting the novel classification of AC as a multicellular and multiorgan disease.

## 1. Introduction

Arrhythmogenic cardiomyopathy (AC) is a familial disorder in the young and athletes, representing one of the main causes of sudden cardiac death (SCD) due to arrhythmic events. Among patients with genetically identifiable cause, about 50% harbor mutations in genes encoding desmosomal proteins. The hallmark of the AC myocardium is its replacement with fibro-fatty tissue, which affects both cardiac electrical activation and contractile function, leading to stress-related life-threatening arrhythmias and possible progression to heart failure (HF) [1,2,3,4]. In many cases, cardiac arrest follows the sympathetic surge that occurs during acute exercise, which has been shown to independently accelerate disease progression [5,6]. The spectrum of AC phenotypes includes variants with right- (ARVC), left- (ALVC), and bi-ventricular (AC) remodeling, which segregate with specific genotypes. AC is still an orphan of mechanism-driven efficient therapies as its pathogenesis is unclear [7,8,9]. 

We believe that the common misconception that AC is a desmosome-related disease has focused researchers’ efforts primarily on desmosome-carrying cells (i.e., cardiomyocytes, CMs). However, current literature and our data show that desmosomal proteins are expressed by other cardiac cells, which would thus all harbor the respective pathogenetic variant in AC hearts and participate in disease pathogenesis. Among these, cardiac mesenchymal stromal cells (C-MSCs) were demonstrated to have cell-autonomous propensity to differentiate into adipocytes and contribute to the adipose deposition in human AC myocardium [10]. However, the extent of the fibro-fatty lesions cannot be explained by the limited number of resident C-MSCs, and thus the potential contribution of extra-cardiac MSCs to myocardial remodeling, which has not previously been accounted for so far, cannot be ruled out.

It is important to remind that MSCs are present not only in the heart, which is the targeted organ in AC, but also in several extra-cardiac districts. Among these, the bone marrow (BM) is one of the main sources of MSCs [11,12,13], but its potential role in AC has never been addressed. The fact that desmosomal variants are germline mutations, and the evidence accrued by us that both cardiac- and BM- MSCs express desmosomal proteins, imply that MSCs in both compartments may be affected in AC. Here, we tested the novel hypothesis whereby AC, although dramatically manifesting with the sudden heart short-circuit, develops with the contribution of multiple cell types and systems. To this purpose, we compared cardiac- vs. BM- MSCs from control and homozygous desmoglein-2 (*Dsg2*) mutant mice, a preclinical model of AC [14]. In detail, these mice carry a loss-of-function mutation in murine *Dsg2* due to the excision via Cre-mediated recombinase mating of exons 4 and 5 in the *Dsg2* gene, located in chromosome 18 (*Dsg2*^mut/mut^ mice). Such excision causes a frameshift mutation, generation of two stop codons in Exon 6, and premature truncation of mRNA, which is then degraded, leading to *Dsg2* protein downregulation. *Dsg2*^mut/mut^ mice well replicate the disease phenotype observed in a significant fraction of AC patients [14,15]. In addition, this mouse model is well-suited for this research scope, as it carries the AC-linked mutation in all *Dsg2* expressing cells, including MSCs, thus mimicking the germline expression of the disease-linked gene variants in humans. 

## 2. Materials and Methods

### 2.1. Animal Models

In this study, we used male *Dsg2*^mut/mut^ mice [14] and age- and sex-matched littermate controls at different ages (2 weeks, 3 and 4 months (mo.)). Animals were maintained in an authorized animal facility (authorization number 175/2002A) at a controlled temperature with a 12-on/12-off light cycle and had access to water and food ad libitum. All experimental procedures performed on rodents were approved by the local ethical committee and the Ministry of Health (authorization numbers 408/2018PR approved in 11 June 2018 and 129/2018PR approved in 13 February 2018), in compliance with Italian Animal Welfare Law (Law n 116/1992 and subsequent D.Lgs. 26/14). All procedures were performed by trained personnel with documented formal training and previous experience in experimental animal handling and care. All procedures were refined prior to starting the study, and the number of animals was calculated to use the least number of animals sufficient to achieve statistical significance according to sample power calculation.

### 2.2. Isolation of Mesenchymal Stromal Cells (MSCs) from Murine Hearts

MSCs were isolated from the hearts of young (2 weeks) and adult (4 months) control and AC *Dsg2*^mut/mut^ mouse hearts by using the protocol described by Ackers-Johnson and colleagues [16]. Cardiomyocytes were divided from non-myocyte cells by centrifugation. Among the pool of cardiac interstitial cells, MSCs were selected, firstly, based on their adherence to plastic, and, subsequently, by flow cytometry screening (FACS Canto II [BD Biosciences, San Jose, CA, USA]), using a panel of MSC surface markers (Supplementary Table S1), according to Dominici et al. [17]. Cells were cultured as described in Angioni et al. [18]. 

### 2.3. Isolation of MSCs from Murine Bone Marrow (BM)

BM-MSCs were isolated from 2-week-old and 4-month-old *Dsg2*^mut/mut^ mice and age- and sex-matched littermate controls, using the protocol described in Angioni et al. [18]. BM-MSCs were selected based on adherence to plastic and were screened by flow cytometry, according to Dominici et al. [17]. 

### 2.4. RTqPCR

Analysis was performed as previously described in Zaglia et al. [19]. The oligos used in this study are listed in Supplementary Table S2.

### 2.5. Western Blotting (WB)

Proteins were extracted from cultured control and *Dsg2^mut/mut^* cardiac- and BM-MSCs, using the lysis buffer, described in Muinao et al. [20]. WB was then performed as described in Zaglia et al. and Muinao et al. [19,20]. Primary antibodies used in this study are listed in Supplementary Table S3. Densitometry was calculated using Fiji software [ImageJ, US NIH, USA] [21]. 

### 2.6. Viral-Assisted Dsg2 Silencing in Normal Rat Cultured BM-MSCs

Normal rat BM-MSCs were transduced with an adenoviral (Ad) vector coding an shRNA targeting *Dsg2* (sh*Dsg2*) (Ad-mCherry-U6-r-*Dsg2*-shRNA, Vector Biolabs) at different concentrations (i.e., 25, 50, and 100 MOI (multiplicity of infection)). Scrambled Adenovirus (Ad-Empty) was used as control. Transduction was validated by WB. Cells were used for in vitro and morphometric assays. 

### 2.7. Morphometric Analyses of Cultured MSCs

Images of cardiac- and BM-MSCs, stained with AlexaFLUOR^®^-568 conjugated phalloidin [Thermo Fisher Scientific, Waltham, MA, USA], were acquired using a ZEISS LSM900 confocal microscope [Carl Zeiss, Oberkochen, Germany]. Cell area, perimeter, and perimeter/area ratio were evaluated by manually drawing regions of interest and calculating parameters, using Fiji software [ImageJ, US NIH, USA] [21].

Images of cells stained with anti-α-tubulin were acquired with the super-resolution mode of the ZEISS LSM900 Airyscan II [Carl Zeiss, Oberkochen, Germany] and processed by sequential binarization and skeletonization, which were used to increase the signal-to-noise ratio of α-tubulin fluorescence and to allow for automatic quantitation using Fiji software [21].

### 2.8. Transwell Migration Assay

Migration assay was carried out in a 24-well Transwell using polycarbonate membranes with 8 μm pores (Falcon). Control and AC cardiac- and BM-MSCs (100.000 cells in 200 μL of serum-free medium (Dulbecco’s Modified Eagle’s Medium (DMEM) -Low Glucose [Lonza, Basel, Switzerland] +2 mM P/S [Lonza, Basel, Switzerland] L/G [Gibco, Thermo Fisher Scientific, Waltham, MA, USA]) were placed in the upper chamber of the Transwell assembly. The lower chamber contained 600 μL of the same medium. Medium supplemented with 10% fetal bovine serum (FBS) (Labtech, Sorisole, Italy) was used as positive control. MSCs were allowed to migrate for 18 h at 37 °C in 5% CO_2_. Then the membranes were fixed in Methanol (Sigma-Aldrich, St. Luis, MO, USA), and the bottom side was stained with 0.1% Crystal Violet for 10 min at room temperature. Two images per insert were acquired with ZEISS AxioImager M1brightfield color microscope [Carl Zeiss, Oberkochen, Germany], and analysis was performed using Fiji software [ImageJ, US NIH, USA] [21]. 

### 2.9. In Vivo Competitive Homing Assay

BM-MSCs from *Dsg2*^mut/mut^ and control mice were labelled with Qtracker^®^ 525 or Qtracker^®^ 655, using the cell labelling kit (Qtracker™ 525 and 655 Cell Labelling Kit [Thermo Fischer Scientific, Waltham, MA, USA]), following the manufacturer’s instructions. Once the efficacy of cell labelling was assessed at the flow cytometry, a pool of control and AC cells (0.25 × 10^5^ AC plus 0.25 × 10^5^ control cells) was resuspended in 100 μL of sterile PBS and injected, via tail vein, in 3 mo. control or *Dsg2*^mut/mut^ mice. Mice were sacrificed 24 h after cell injection and different organs (i.e., heart, lungs, liver, and spleen) were harvested. Heart, lungs, and liver were digested in Hanks Balanced Salt solution (HBSS) [Lonza, Basel, Switzerland], supplemented with 5% FBS [Gibco, Thermo Fisher Scientific, Waltham, MA, USA], 10 mM HEPES [Lonza, Basel, Switzerland], 1.5 mg/mL collagenase II [Gibco, Thermo Fisher Scientific, Waltham, MA, USA], and 0.4mg/mL DNase I [Roche, Basel, Switzerland]. The spleen was mechanically dissociated on a 70 µm nylon filter [Falcon, Corning, Tewksbury, MA, USA] in PBS. From the pool of isolated cells, red blood cells were lysed in ACK lysing buffer [Lonza, Basel, Switzerland], and the remainder cells were resuspended in PBS for subsequent FACS analysis. Non-viable cells were excluded after Aqua (Invitrogen Corporation, Carlsbad, CA, USA) staining, and the fraction of MSCs was gated according to Dominici et al. [17]. The amount of fluorescent labelled BM-MSCs was calculated as a fraction over the total number of events from different organs. Data were analyzed with FlowJo [FlowJo LLC, Ashland, OR, USA]. The total amount of fluorescent labelled cells was plotted.

### 2.10. Statistical Analysis

The sample size per group was estimated with sample power analysis using previously determined standard deviations in similar experiments. Data were analyzed using Prism Software (GraphPad, La Jolla, CA, USA). Gaussian distribution of data was assessed before performing statistical analysis by applying Shapiro–Wilk normality test. Data were since expressed as either ‘mean±SD (standard deviation)’ or ‘median with 95% confidence interval (CI)’, depending on the normality of the data distribution. Differences among groups were evaluated by parametric (in normally distributed samples: *t*-test with or without Welch’s correction; one-way ANOVA with Tukey’s multiple comparison test) or non-parametric tests (i.e., Mann–Whitney test; Kruskal–Wallis test with Dunn’s multiple comparison test; two-Way ANOVA with Sidak’s multiple comparison test). Differences with a *p*-value lower than 0.05 were considered significant.

## 3. Results

### 3.1. Cardiac and Bone Marrow Mesenchymal Stromal Cells Express Desmosomal Proteins

**Figure 1:**
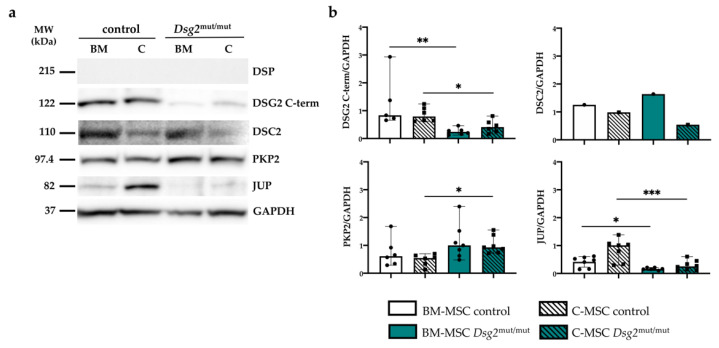
Cardiac and bone marrow mesenchymal stromal cells express desmosomal proteins, whose profile changes in the AC background. (**a**) Western blotting on protein extracts from cultured cardiac- and BM-MSCs, isolated from either control or *Dsg2*^mut/mut^ adult male mice. GAPDH was used to ensure equal protein loading. MW, molecular weight. DSP, desmoplakin; DSG2, desmoglein-2; DSC2, desmocollin-2; PKP2, plakophilin-2; JUP, plakoglobin. (**b**) Densitometric analyses of the WB in (**a**). A total of 4 samples for each group were analyzed. Three independent experiments were performed, with the exception of DSC2. In the graph of JUP, bars represent mean, and error bars represent the relative standard deviation (SD). In the others, bars represent median, and error bars represent the limits of 95% confidence interval (CI). * *p* ≤ 0.05; ** *p* ≤ 0.01; *** *p* ≤ 0.001.

It has been demonstrated that C-MSCs from human heart biopsies express desmosomal proteins [10], thus indicating that the cardiac mesenchymal cell compartment is also affected in AC. Whether this holds true also in the mouse heart, a widely used model in AC preclinical studies, has never been investigated. In addition, whether desmosomal proteins are also expressed by extra-cardiac MSCs—which may thus add to the spectrum of diseased cells in AC—has never, to the best of our knowledge, been evaluated. To address this point, here we compared the expression of desmosomal genes in cultured MSCs isolated from heart (the main target organ in AC) and BM (one of the main sources of MSCs) from control vs. *Dsg2*^mut/mut^ adult mice. Cardiac- and BM-MSCs were isolated, as described in the Materials and Methods section, and characterized based on the expression of a panel of MSC surface markers [17] (Supplementary Figures S1 and S2). Our analysis shows that, in the normal genetic background, both cardiac- and BM-derived MSCs expressed desmosomal genes at similar levels, with the exception of plakoglobin (*Jup*), which was higher in cardiac-, compared with BM-derived MSCs (Supplementary Figure S3). Notably, all desmosomal proteins were detectable by WB, except desmoplakin (DSP), likely due to its very low protein level (Figure 1a). In AC cells, DSG2 protein content was, as expected [14], significantly downregulated in both cardiac- and BM-MSCs (Figure 1a,b). Remarkably, expression of the AC-linked *Dsg2* gene variant was accompanied with increased PKP2 protein level in C-MSCs and decreased JUP, compared with controls, in both heart and BM-derived cells (Figure 1a,b). The previous evidence that C-MSCs have cell-autonomous propensity to differentiate into adipocytes in AC [10] and our finding of modification of the desmosomal protein profile in AC MSCs, harboring the *Dsg2* variant, further support that these cells may be implicated in the disease mechanism and warrant further research on this cell type. In addition, the finding that BM-MSCs share, with the cardiac counterpart, similar gene and protein expression surmises that the list of mesenchymal cell types participating in AC also includes those from extra-cardiac compartments.

### 3.2. Dsg2^mut^^/mut^ Cardiac and Bone Marrow Mesenchymal Stromal Cells Show Cytoskeletal Alteration

**Figure 2:**
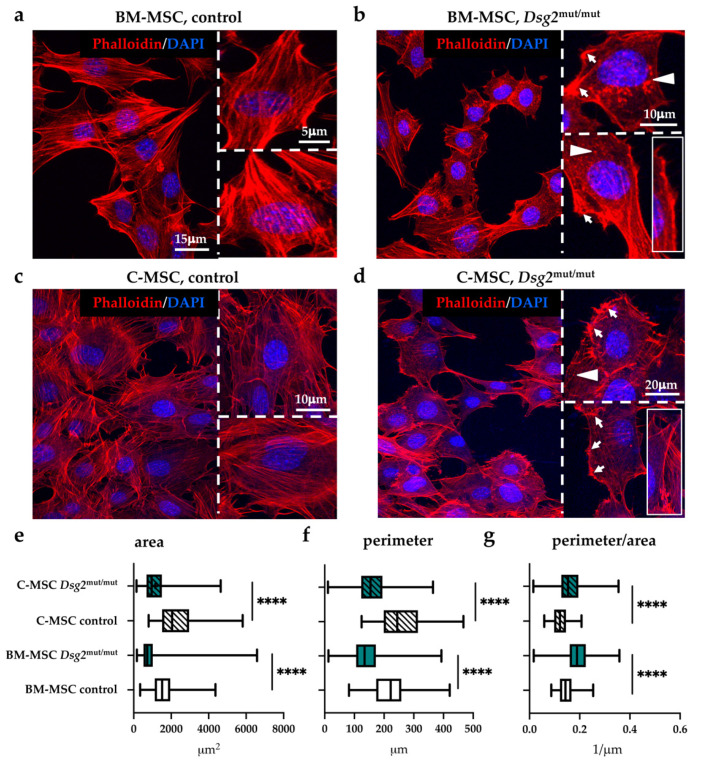
AC-linked *Dsg2* variant affects cytoskeletal organization and morphology of cardiac and bone marrow mesenchymal stromal cells. (**a**–**d**) Confocal IF analysis of cultured cardiac- and BM-MSCs isolated from control or *Dsg2*^mut/mut^ mice. Cells were stained with AlexaFLUOR^®^-568 conjugated phalloidin (red signal). Nuclei were counterstained with DAPI (blue signal). White arrows indicate focal adhesions, and arrowheads evidence actin puncta. Insets show sub-membrane thick actin filaments. (**e**–**g**) Morphometric evaluation of cell surface, perimeter, and perimeter/area ratio in cultured cardiac and BM-MSCs isolated from control or *Dsg2*^mut/mut^ mice. A total of 110 cells for each study group were analyzed. Whiskers represent min to max values. **** *p* ≤ 0.0001.

To assess how *Dsg2*-linked AC impacts on MSCs, we first compared the morphologic features of cells isolated from either the heart or BM of adult control and AC mice. At a first inspection with bright-field microscopy, we noticed that the AC mutant population included numerous grossly rounded and less frequent spindle-shaped cells, compared with controls, with a decrease in dimension. Based on the well-accepted role of the cytoskeletal dynamics and organization in determining cell shape, spreading, and stiffness, which eventually affect cell differentiation [22], and on the role of DSG2 in regulating actin assembly [23,24,25], cells were initially analyzed with confocal microscopy upon incubation with AlexaFLUOR^®^-568 conjugated phalloidin (see Supplementary Methods) to determine the distribution of intermediate filaments. Our analysis revealed that in the vast majority of *Dsg2*^mut/mut^ cells, the actin cytoskeleton—which in control cells, displayed an abundant number of thin, parallel microfilament bundles, extending across the entire cytoplasm—was dramatically different (Figure 2a–d). In fact, both *Dsg2*^mut/mut^ BM- and cardiac- MSCs showed alterations in actin distribution, characterized by the presence of cytoplasmic regions devoid of organized filaments with fluorescent puncta, regions occupied by thick actin filament bundles, predominantly located at the outermost cell periphery, and membrane localized focal adhesions, all of which reminisce actin stress fibers and suggest alterations in microfilament dynamics (Figure 2a–d). In addition, both BM- and cardiac- AC cells were smaller in size (surface area, BM-MSC, *Dsg2*^mut/mut^: 938.8 ± 824.5 vs. control: 1611 ± 676; C-MSC, *Dsg2*^mut/mut^: 1157 ± 709.3 vs. control: 2330 ± 1138, in μm^2^), had a significantly shorter perimeter (BM-MSC, *Dsg2*^mut/mut^: 150.9 ± 69.34 vs. control: 220.1 ± 61.62; C-MSC, *Dsg2*^mut/mut^: 162.3 ± 57.61 vs. control: 258 ± 74.99, in μm), and a higher perimeter/area ratio (BM-MSC, *Dsg2*^mut/mut^: 0.1921 ± 0.05787 vs. control: 0.1466 ± 0.03411; C-MSC: *Dsg2*^mut/mut^: 0.1638 ± 0.05423 vs. control: 0.1232 ± 0.03432) (Figure 2e–g). This latter result was used as an indicator of general modifications in the cell shape.

**Figure 3:**
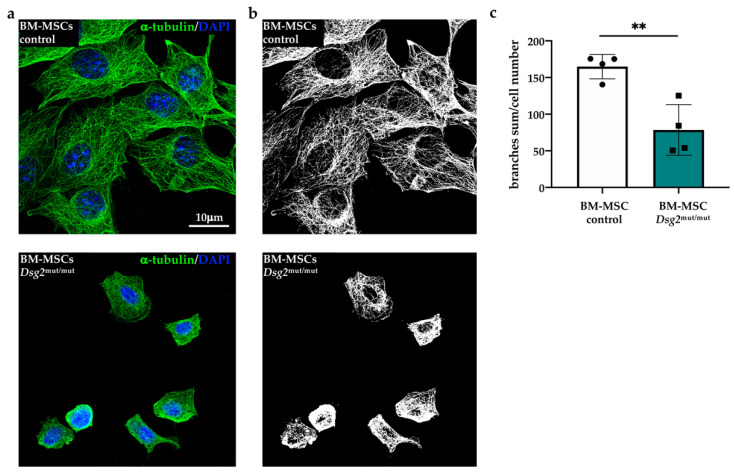
AC-linked *Dsg2* variant affects microtubule organization in bone marrow mesenchymal stromal cells. (**a**) Super-resolution confocal IF analysis of cultured BM-MSCs isolated from control or *Dsg2*^mut/mut^ mice. Cells were stained with anti-α-tubulin antibody (green signal). Nuclei were counterstained with DAPI (blue signal). (**b**) Skeletonized images shown in (**a**). (**c**) Quantification of total number of microtubule branches/cell in control and AC BM-MSCs. A total of 30 cells for each study group were analyzed. Bars represent mean, and error bars represent standard deviation (SD). ** *p* ≤ 0.01.

**Figure 4:**
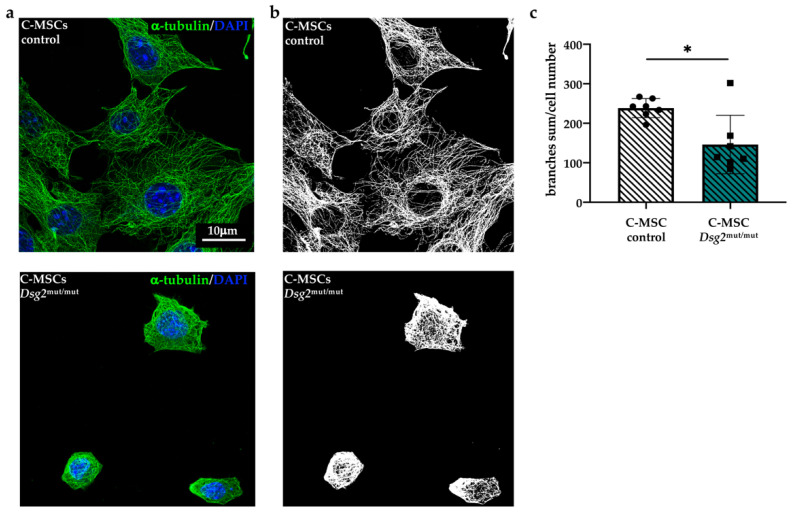
AC-linked *Dsg2* variant affects microtubule organization in cardiac mesenchymal stromal cells. (**a**) Super-resolution confocal IF analysis of cultured C-MSCs isolated from control or *Dsg2*^mut/mut^ mice. Cells were stained with anti-α-tubulin antibody (green signal). Nuclei were counterstained with DAPI (blue signal). (**b**) Skeletonized images shown in (**a**). (**c**) Quantification of total number of microtubule branches/cell in control and AC C-MSCs. A total of 35 cells for each study group were analyzed. Bars represent mean, and error bars represent standard deviation (SD). * *p* ≤ 0.05.

To gain additional details on cytoskeletal organization, we imaged microtubules in cultured cells stained with an anti-α-tubulin antibody. Analysis at confocal and super-resolution microscopy revealed that, in addition to microfilaments, the microtubule network was also altered in AC cells. Alpha-tubulin filaments, which had a regular distribution and covered most of the cell cytoplasm in control cells, appeared irregularly distributed, partially fragmented, and threaded around the cell nucleus in both BM-MSCs (Figure 3a,b) and C-MSCs (Figure 4a,b) (Supplementary Figure S4). Unbiased analysis of the total number of branches quantitated such qualitative alterations in the microtubular network, which appeared less branched in the AC cells (Figure 3c and Figure 4c). Taken altogether, our results, by confirming previous evidence on the role of DSG2 in regulating cytoskeleton of different cell types [22,23,24,25], indicate that the primary *Dsg2* mutation may affect the biology of both cardiac- and BM-MSCs. 

### 3.3. DSG2 Downregulation Affects Mesenchymal Stromal Cells Cytoskeletal Organization

**Figure 5:**
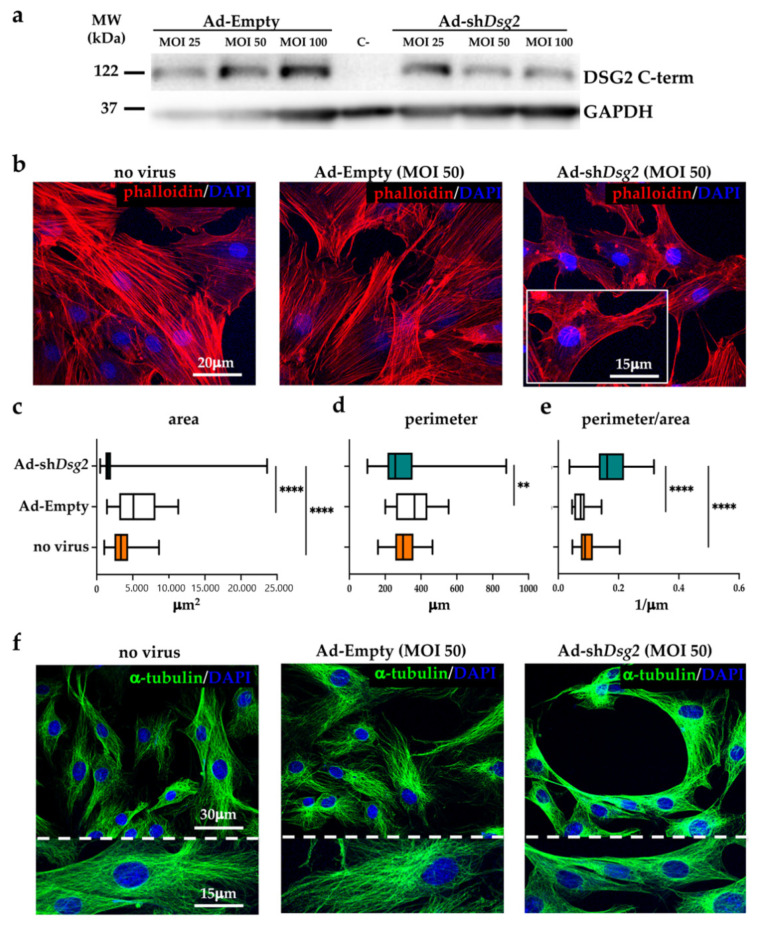
DSG2-downregulated bone marrow mesenchymal stromal cells display altered cytoskeletal organization. (**a**) Western blotting on protein extracts from cultured rat BM-MSCs transduced with either Ad-Empty or Ad-sh*Dsg2* at different MOI (25, 50, and 100). GAPDH was used to ensure equal protein loading. MW, molecular weight. C-, negative control (*Dsg2^mut/mut^* heart). (**b**) Confocal IF analysis of cultured rat BM-MSCs infected with Ad-Empty (MOI 50) or Ad-sh*Dsg2* (MOI 50). Cells were stained with AlexaFLUOR^®^-568 conjugated phalloidin (red signal). Nuclei were counterstained with DAPI (blue signal). (**c**–**e**) Morphometric evaluation of cell surface, perimeter, and perimeter/area ratio in cultured rat BM-MSCs shown in (**b**). A total of 25 cells for each study group were analyzed. Whiskers represent min to max values. ** *p* ≤ 0.01; **** *p* ≤ 0.0001. (**f**) Confocal IF analysis of cultured rat BM-MSCs infected with Ad-Empty (MOI 50) and Ad-sh*Dsg2* (MOI 50). Cells were stained with anti-α-tubulin antibody (green signal). Nuclei were counterstained with DAPI (blue signal).

The results of the experiments described thus far suggest that the AC-linked *Dsg2* variant impinges on the regulation of MSC cytoskeletal dynamics and function. To determine whether the AC cell phenotype is due to a deficiency in DSG2 protein level, as a result of *Dsg2* mRNA-mediated degradation in *Dsg2*^mut/mut^ mice [14,15], we conjectured that DSG2 downregulation would lead to the same alterations in normal rat BM-MSCs. To test this hypothesis, BM-MSCs were infected for 72 h with an adenoviral (Ad) vector encoding a short hairpin RNA (sh-RNA), which binds to *Dsg2* mRNA and prevents protein translation. Rat cells were chosen for the abundance of material harvested from a single animal, which allowed reduction of the number of experimental animals used in this work. In addition, based on the similarity between mouse and rat BM-MSC desmosomal profile, we decided to verify our hypothesis in another species. Three days after infection, DSG2 protein levels were assessed by WB, confirming the expected reduction in DSG2 protein level, which was of similar extent in cells inoculated with either MOI 50 or 100 (85.1 ± 5.6 % reduction compared with controls, *n* = 3 independent experiments; *p* ≤ 0.01) (Figure 5a). We then analyzed with confocal microscopy, in phalloidin-labelled cells, the organization of actin intermediate filaments. As shown in Figure 5b, DSG2 reduction caused both actin filament remodeling and alterations in cell shape, which replicated the morphology of MSCs isolated from *Dsg2*^mut/mut^ mice. In fact, DSG2-downregulated cells were smaller (surface area: Ad-sh*Dsg2*: 2362 ± 3327 vs. Ad-Empty: 5641 ± 2837, in μm^2^; perimeter: Ad-sh*Dsg2*: 290.8 ± 129.8 vs. Ad-Empty: 361.7 ± 109.1, in μm) (Figure 5c,d) and had a higher perimeter/area ratio (Ad-sh*Dsg2*: 0.1709 ± 0.06182 vs. Ad-Empty: 0.07346 ± 0.02321) (Figure 5e). In addition, Ad-sh*Dsg2* cells showed alterations in the intracellular organization of microtubules, which were fragmented and threaded around the cell nucleus (Figure 5f), recapitulating the phenotype described in *Dsg2*^mut/mut^ cells. Thus, these data identify the role of the sole reduction in DSG2 levels and unveil the previously neglected effect of DSG2 in BM-MSCs, supporting a causal link between AC-linked loss-of-function *Dsg2* variant and altered cytoskeletal organization in MSCs. 

### 3.4. AC-linked Dsg2 Variant Affects Proliferation Rate and Migratory Capacity of Cardiac and Bone Marrow Mesenchymal Stromal Cells, In Vitro

**Figure 6:**
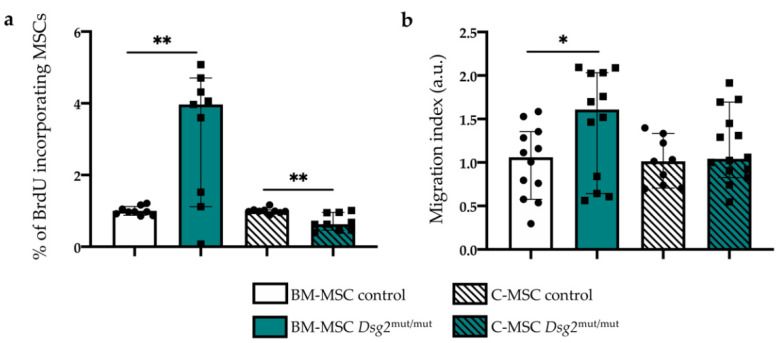
AC-linked *Dsg2* variant enhances proliferation rate and migratory capacity of bone marrow mesenchymal stromal cells in vitro. (**a**) In vitro BrdU incorporation assay in control and *Dsg2*^mut/mut^ cardiac- and BM-MSCs. The fraction of BrdU incorporating MSCs was quantitated. At least 15.000 events per sample were acquired. Three independent experiments were performed. Bars represent mean, and error bars represent standard deviation (SD). ** *p* ≤ 0.01. (**b**) Transwell migration assay in cultured control and *Dsg2*^mut/mut^ cardiac- and BM-MSCs. Two independent experiments were performed. Bars represent median, and error bars represent the limits of 95% confidence interval (CI). * *p* ≤ 0.05.

In light of the well-accepted role played by the microtubule and microfilament network in the regulation of cell proliferation and migration [22,23,24,25,26,27,28,29], the finding of altered cytoskeletal architecture in AC cells prompted us to verify whether the proliferative and migratory capacity of MSCs, harboring the *Dsg2*-variant, differed from controls. We thus estimated in vitro the proliferative index of AC vs. control MSCs, of either cardiac or BM origin, by performing a BrdU incorporation assay and measured the fractions of cells immunoreactive toward an anti-BrdU antibody via flow cytometry. While *Dsg2*^mut/mut^ BM-MSCs showed increased proliferation rate compared with control cells, C-MSCs displayed, conversely, lower BrdU incorporation (Figure 6a). Subsequently, we used a Transwell assay to compare the cell motility capacity of the different cell types. In basal conditions, *Dsg2*^mut/mut^ BM-derived cells displayed increased migratory behavior (Figure 6b), which increased at a level comparable with that of control cells, in the presence of FBS (not shown), to evoke the maximal migratory stimulation. Such differences were not detected in C-MSCs from both genotypes, which showed a comparable migratory capacity, both in basal and stimulated conditions (Figure 6b).

### 3.5. In Vivo Quantitation of Proliferative Index and Content of Cardiac vs. Bone Marrow Mesenchymal Stromal Cells, during AC Progression

**Figure 7:**
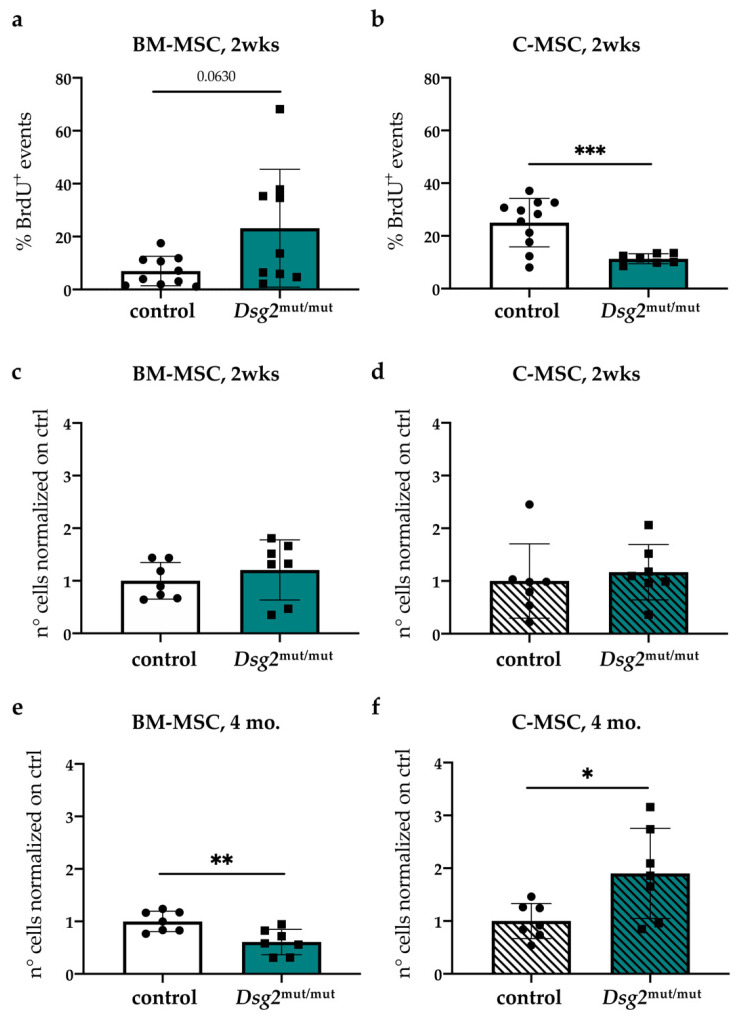
Cardiac and bone marrow mesenchymal stromal cell compartments are affected in *Dsg2*^mut/mut^ mice. (**a**–**b**) In vivo BrdU incorporation assay in young control vs. *Dsg2*^mut/mut^ mice. The fraction of BrdU incorporating BM- (**a**) and cardiac- (**b**) MSCs was quantified by flow cytometry. Bars represent mean, and error bars represent standard deviation (SD). *** *p* ≤ 0.001. (**c**–**f**) Quantification of cardiac- (**d,f**) and BM- (**c,e**) MSC content, evaluated in young (2 wks) (**c**–**d**) and adult (4 mo.) (**e**–**f**) control and AC mice. Bars in (**c,f**) represent mean, and error bars represent standard deviation (SD). *n* = 7 mice per condition. * *p* ≤ 0.05; ** *p* ≤ 0.01.

The evidence accrued in vitro that the AC-linked *Dsg2* mutation impacts on MSC biology prompted us to determine whether the same alterations were detectable in vivo. We thus treated young (2 weeks) control and AC mice with BrdU, as described in the Methods and Materials section. We chose such a time point to exclude cell proliferation due to active cardiac interstitial remodeling. In line with in vitro results, BM-MSCs tended to have increased proliferation rate (Figure 7a), which was significantly lower in the heart-derived cells (Figure 7b). To get insight into the dynamic behavior of MSCs during disease development, we quantitated the relative content of cardiac- and BM-MSCs by flow cytometry in *Dsg2*^mut/mut^ and control male mice at two weeks of age, when the disease is still at subclinical stage, and at 4 months, when structural and functional cardiac alterations are evident [14] (Supplementary Figure S5a–d). Among total cardiac interstitial and BM cells, the MSC fraction was analyzed by flow cytometry, selecting cells according to a panel of widely accepted markers [17], as detailed above. At 2 weeks of age, the number of MSCs of *Dsg2*^mut/mut^ and control mice was comparable between cohorts in both BM and heart (Figure 7c–d). However, at 4 months, in parallel with the diffuse cardiac fibrotic remodeling [14], the number of MSCs in hearts significantly increased, while it decreased in BM of *Dsg2*^mut/mut^ mice, compared with controls (Figure 7e–f). 

**Figure 8:**
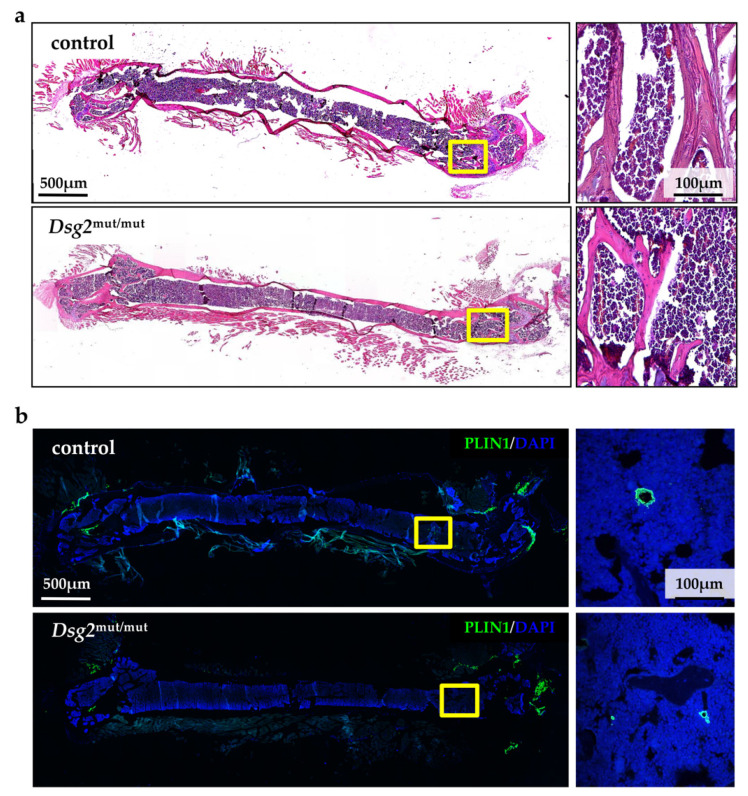
AC-linked *Dsg2* variant does not alter bone marrow structure. (**a**) Hematoxylin-eosin staining of femur BM sections from adult control (top panel) and *Dsg2*^mut/mut^ (bottom panel) mice. The right panels are high magnifications of the yellow boxes in the left ones. (**b**) IF analysis of femur BM sections from adult control (top panel) and *Dsg2*^mut/mut^ (bottom panel) mice, stained with an antibody to perilipin-1 (PLIN1). Nuclei were counterstained with DAPI. The right panels are high magnifications of the yellow boxes in the left ones.

Hematoxylin-eosin staining and IF analysis with anti-perilipin-1 (PLIN1) antibody ruled out that such a finding depended on structural alterations in the BM of adult *Dsg2*^mut/mut^ mice (e.g., fatty accumulation), which could lead to depauperation of MSC niches (Figure 8). 

Taken together, in vitro and in vivo results demonstrate that the AC-linked *Dsg2* mutation impacts on fundamental characteristics of the MSC population, remarkably with specific differences depending on the cellular subtype. At time points coinciding with the largest amount of myocardial damage, the finding that *Dsg2*^mut/mut^ hearts included more MSC than controls was in line with a mechanism combining (i) activation of resident cardiac cells and (ii) possible recruitment of BM cells to the site of damage.

### 3.6. Intravenously Injected Bone Marrow-Derived Dsg2^mut/mut^ Mesenchymal Stromal Cells Have Increased Propensity to Migrate to the AC Myocardium

**Figure 9:**
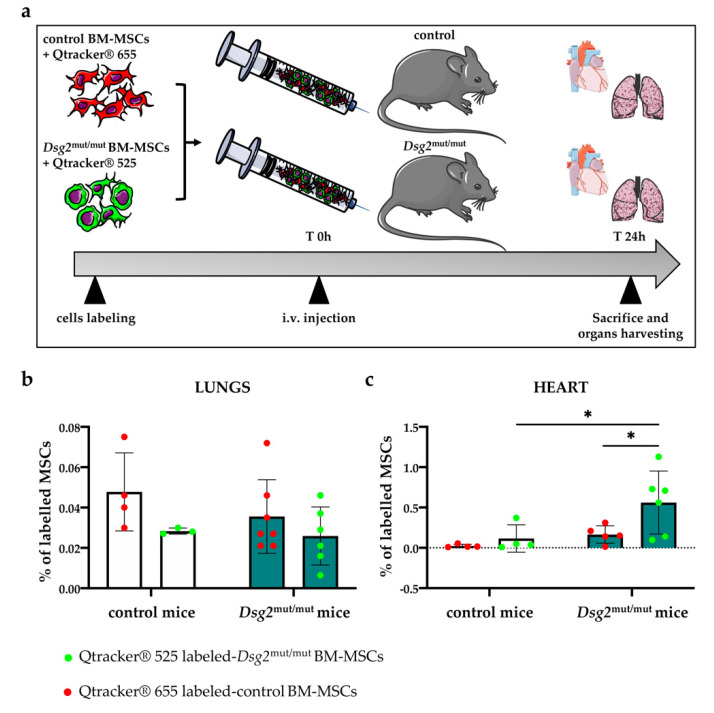
*Dsg2*^mut/mut^ bone marrow-derived mesenchymal stromal cells show increased migration propensity to the AC heart. (**a**) Schematic representation of in vivo competitive homing assay. (**b**,**c**) Quantification, by flow cytometry, of the percentage of fluorescent labelled control and *Dsg2*^mut/mut^ BM-MSCs with respect to the total number of viable MSCs isolated from control or AC lungs (**b**) or hearts (**c**). Bars represent mean, and error bars represent standard deviation (SD). *n* = 4 control mice and *n* = 6 AC mice. * *p* ≤ 0.05.

Our results showing that the reverse changes in the BM and heart MSC content during AC progression, together with the evidence that BM cells actively contribute to heart remodeling in different disease conditions [30,31], guided the hypothesis that engraftment of BM-derived MSCs could play a role in myocardial remodeling in *Dsg2*^mut/mut^ mice. We thus set out to quantify myocardial infiltration by MSCs and to determine whether it was dependent on heart damage, or if the cell intrinsic properties of *Dsg2*^mut/mut^ MSCs could play a role. To this aim, we performed a ‘competitive’ homing assay, in which a balanced mixture of control and *Dsg2*^mut/mut^ BM-MSCs, isolated from adult mice, were labelled in vitro with 655 nm and 525 nm emitting fluorescent Qtrackers^®^ [Thermo Fischer Scientific, Waltham, MA, USA], respectively, and then injected intravenously (i.v.) in adult control and *Dsg2*^mut/mut^ mice. Labelling was very efficient, as more than 95% of cells were positively colored before injection (Supplementary Figure S6). After 24 h, mice were euthanized and lungs (which are known to accumulate cells injected i.v. as result of pulmonary first-pass) [32], liver, spleen, and heart were collected (Figure 9a). Organs were minced and enzymatically digested to obtain a single cell suspension, which was further processed with flow cytometry. There were no genotype-dependent differences in MSC accumulation in the lungs (Figure 9b) and liver, which showed that all animals received similar amounts of labeled cells; there was only a moderate decrease in MSC in the spleen of AC mice. However, AC hearts had increased MSC accumulation than controls (Figure 9c), and remarkably, the majority of infiltrating cells originated from the *Dsg2*^mut/mut^ pool. These differences were not artifactual, as shown by the same results obtained upon injection of the mixture of control and AC BM-MSCs, labelled with inverted colors (655 nm *Dsg2* mutant vs. 525 nm control cells). 

These results are consistent with the effect of tissue damage, which, likely through the release of chemoattracting factors, recruits circulating MSCs to the site of damage. Interestingly, these data also suggest that BM-MSCs, harboring the *Dsg2* variant, have a higher propensity to migrate and engraft the isogenic AC heart. This concept was emphasized by the finding that, upon independent injections of either normal or *Dsg2*^mut/mut^ labeled BM-MSCs in AC mice—despite both control and *Dsg2*^mut/mut^ cells infiltrating the lungs at similar levels—the latter were found in larger number in hearts (Supplementary Figure S7). In a subset of experiments, hearts were removed and fixed to seek whether labelled BM-MSCs accumulated preferentially in specific (e.g., damaged) areas. Unfortunately, due to the very low number of cells trapped in the whole myocardium, we were not able to detect fluorescent cells in thin heart sections. However, our results indicate that, independent from the presence of tissue damage, *Dsg2*^mut/mut^ BM-derived MSCs have increased propensity to migrate to the AC heart, supporting the notion whereby BM-derived MSCs may contribute to the cardiac fibro-fatty scars.

## 4. Discussion

Arrhythmogenic cardiomyopathy (AC) is a familial cardiac disorder featuring CM death, tissue inflammation, and myocardial fibro-fatty remodeling, typically starting from the right ventricle epicardium, accounting for most cases of SCD in the young and athletes [3,7]. In about 50% of genetically diagnosed cases, AC is caused by mutations in genes encoding desmosomal proteins [3,7], and desmosomes, which form strong cell-to-cell adhesion structures, allowing mechanically connected cardiomyocytes (CMs) to sustain contractile stress, have attracted most attention. In this work, we show that in cardiac mesenchymal stromal cells (C-MSCs), which natively express desmosomal proteins and have previously been implicated in myocardial remodeling in AC [10], expression of an AC-linked variant of *Dsg2* affected cell replication rate and morphology. Furthermore, our study extends the analysis to MSCs in other compartments, such as the bone marrow (BM), and demonstrates that cells harboring the *Dsg2* variant have increased motility and potential to engraft the injured heart and that they are mobilized and likely homed in the AC heart during progression of myocardial remodeling. Our experiments suggest that all these effects may depend on cytoskeletal alterations, which were detected in MSCs from *Dsg2*^mut/mut^ mice and replicated by DSG2 downregulation in normal cells. 

The mechanisms underlying myocardial remodeling in AC are in urgent need of understanding, because if fibro-fatty scars compromise the correct cardiac mechanical and electrical activity, counteracting heart remodeling may prevent cardiac dysfunction and fatal arrhythmias. The study from Sommariva et al. [10] showed that C-MSCs contribute to the formation of the fatty myocardial lesions identified in human AC hearts. In the same research, the authors demonstrated that C-MSCs express desmosomal proteins, implying therefore that they carry AC-linked mutations and may also, together with CMs, be affected in AC. 

This prompted our initial research, which demonstrated that almost all cardiac and extra-cardiac cells express desmosomal proteins, independent from forming desmosomes. The role of desmosomal proteins in non-myocyte cells is poorly understood, but given the germline transmission of AC variants, the biology of both cardiac and extra-cardiac cell populations may potentially be affected by the disease-causing mutation. In addition, AC pathogenesis potentially involves an interaction among cardiac and extra-cardiac systems. In further support of this, recently, Agrimi et al. demonstrated that the ‘brain-to-heart’ communication has a role in the disease pathogenesis [33]. In fact, psycho-social stress in AC mice accelerates myocardial remodeling and increases the incidence of SCD, supporting the novel idea that an impaired inter-organ crosstalk may also occur [33]. Thinking of AC as a multicellular and multiorgan disorder, we thus aimed at determining whether other extra-cardiac mesenchymal compartments, such as the BM, could be affected in AC. 

The BM is one of the main sources of MSCs, and myocardial injury and cell death recruit BM-derived MSCs, which contribute to heart remodeling [30,31]. We thus assessed the expression of desmosomal genes in cardiac- and BM-MSCs, compared the levels of the different desmosomal proteins in the two cell populations, and assessed the effects of AC-linked *Dsg2* variant on cardiac- and BM-MSC phenotypes and biology. In line with human data, both cardiac- and BM-MSCs express desmosomal proteins at similar levels, and their profile is altered in the AC genetic background. In fact, DSG2 downregulation was accompanied by increased protein levels of PKP2 in *Dsg2*^mut/mut^ C-MSCs, while JUP content dropped in both cardiac- and BM-mutant cells. Although this aspect is far from the scope of this work, these results suggest that a cross-regulation between desmosomal proteins exists and may explain why the various forms of AC (i.e., ARVC vs. ALVC), depending on different desmosomal gene mutations, may have partially overlapping phenotypes.

Our results are in line with other studies demonstrating that DSG2 and other desmosomal proteins are expressed by non-desmosome forming cells, including endothelial cells [23], where they have been demonstrated to mediate desmosome-independent functions. DSG2 belongs to the family of desmosomal cadherins, which have a key role in several cellular processes, including cytoskeletal organization, migration, and differentiation [22,23,24]. In endothelial cells, DSG2 has been shown to interact, via PKP2 and JUP, with DSP and, consequently, with the network of intermediate filaments [23,24], playing important roles in cell motility, wound healing, and tissue formation through its regulation of actin dynamics [25]. Consistently, re-localization of actin filaments, resulting from DSG2 downregulation, compromises angiogenesis and reduces cell-to-cell adhesion [23,24]. 

In our experiments, DSG2 downregulation was associated, in both cardiac- and BM-MSCs from *Dsg2*^mut/mut^ mice, with profound alterations in the organization of actin filaments with the coexistence of cell areas occupied by actin ‘puncta’ and thick actin filament bundles beneath the cell membrane. In parallel, we also observed profound changes in the organization of microtubules, which have been shown to regulate cell motility and shape [27] and differentiation properties [28,29]. That such alterations were detected in vitro in two different MSC sub-populations and in a cell culture system free from context dependent factors strongly supports a direct role of AC-linked *Dsg2* variant in influencing the MSC phenotype. In further support of this, viral assisted DSG2 downregulation in normal BM-derived MSCs was sufficient to elicit the same cytoskeletal alterations described in AC MSCs isolated from *Dsg2*^mut/mut^ mice. 

We could not test the mechanistic relationship between expression of the *Dsg2* variant and cytoskeletal alterations, which were, however, entirely replicated by DSG2 down-regulation, showing the direct effect of the mutant allele. Reorganization of the actin cytoskeleton has instead been linked to alterations in cell proliferation rate via ERK (extracellular signal-regulated kinase) signaling pathway [22], an effect consistent with the phenotype of BM-derived *Dsg2*^mut/mut^ cells.

Our data indicate, for the first time, that AC associated with a *Dsg2* mutation also affects the BM, an extra-cardiac mesenchymal compartment, warranting further genotype/phenotype correlation studies. In addition, it stimulates the interest toward identifying factors that may contribute to BM-MSC mobilization and cardiac engraftment.

To determine the cellular dynamics in MSC BM and heart compartments, we quantified the content of cardiac- vs. BM-MSCs in both AC and control mice during disease progression from a subclinical stage to an advanced disease stage with manifest myocardial lesions. Interestingly, while at the earlier disease stage the number of MSCs was similar in BM and heart in both genotypes, at advanced disease stages in AC mice, the MSC content was reduced in the BM and significantly higher in the heart. The increased MSCs number in the AC heart may be explained by the active myocardial remodeling associated with the release of cytokines and chemokines observed in *Dsg2*^mut/mut^ mice [34], recruiting circulating and BM-cells. The finding that at a more advanced disease stage the BM-MSC number decreased was suggestive of enhanced mobilization driven by cardiac remodeling in AC mice and prompted us to test the hypothesis that BM-MSCs may be recruited to the damaged myocardium and that they may contribute to tissue remodeling. Given that BM irradiation is ineffective in ablating MSCs [35,36], the simple reconstitution of the BM with labelled donor cells could not be exploited. We thus used an in vivo cell tracking experiment in which a mixture of control and mutant BM-derived stromal cells were injected intravenously in either control of *Dsg2*^mut/mut^ AC mice. The quantification of fluorescent labelled cells in different organs (i.e., lungs, liver, spleen, and heart) after 24 h following cell injection demonstrated that AC cells are found in the damaged heart at higher fraction, compared with control cells, and display increased propensity to home into the myocardium. The inference that the cytoskeletal alterations observed in vitro may reflect on migratory and engraftment capacity in vivo is well supported [22,23,24,25]. 

When placed into the broader context of AC pathogenesis, our results are not in contrast with the effect of AC variants, including the *Dsg2*^mut/mut^ on CMs, which are in fact the cells responsible for the initial myocardial cell damage and the electrophysiologic manifestations in AC. The effect of *Dsg2*^mut/mut^ on the MSCs of both cardiac and BM origin suggests that the cells harboring the AC variant may have increased propensity to contribute to myocardial remodeling and, possibly, fibro-fatty lesion formation. 

## 5. Conclusions

Collectively, our data describe the effect of an AC-linked genetic variant on MSCs, from both cardiac and BM source, and identify the BM-MSCs as a previously neglected cell pool influenced by the disease mutation, which may participate in heart remodeling. These results identify mechanisms that modulate cardiac- and BM-MSCs and reduce myocardial arrhythmogenic remodeling.

## Data Availability

Data available upon request.

## Supplementary Materials

The following are available online at https://www.mdpi.com/article/10.3390/jcm10091871/s1, Supplementary text, including Supplementary Methods and Figure Legends. Figure S1: Flow cytometry characterization of Cardiac Mesenchymal Stromal Cells (C-MSCs); Figure S2: Flow cytometry characterization Bone Marrow Mesenchymal Stromal Cells (BM-MSCs); Figure S3: Cardiac and Bone Marrow Mesenchymal Stromal Cells express desmosomal genes; Figure S4: AC-linked *Dsg2* variant affects microtubule organization in both Cardiac and Bone Marrow Mesenchymal Stromal Cells; Figure S5: AC-linked *Dsg2* variant progressively affects heart morphology; Figure S6: Flow cytometry characterisation of the efficiency of BM-MSC labelling with Qtracker®. Figure S7: *Dsg2*^mut/mut^ Bone Marrow Mesenchymal Stromal Cells preferentially home to the heart, compared to control BM-MSCs. Table S1: List of antibodies used in this study for FACS analyses; Table S2: Oligos used in this study for RTqPCR analysis; Table S3: List of primary antibodies used in this study for IF and WB analyses.

## References

[1] Thiene G., Nava A., Corrado D., Rossi L., Pennelli N. (1988). Right ventricular cardiomyopathy and sudden death in young people. N. Engl. J. Med..

[2] Basso C., Thiene G., Corrado D., Angelini A., Nava A., Valente M. (1996). Arrhythmogenic right ventricular cardiomyopathy: Dysplasia, dystrophy, or myocarditis?. Circulation.

[3] Basso C., Bauce B., Corrado D., Thiene G. (2012). Pathophysiology of arrhythmogenic cardiomyopathy. Nat. Rev. Cardiol..

[4] Asimaki A., Saffitz J.E. (2014). Remodeling of cell-cell junctions in arrhythmogenic cardiomyopathy. Cell Commun. Adhes..

[5] Corrado D., Basso C., Rizzoli G., Schiavon M., Thiene G. (2003). Does sports activity enhance the risk of sudden death in adolescents and young adults?. J. Am. Coll. Cardiol..

[6] James C.A., Bhonsale A., Tichnell C., Murray B., Russell S.D., Tandri H., Tedford R.J., Judge D.P., Calkins H. (2013). Exercise increases age-related penetrance and arrhythmic risk in arrhythmogenic right ventricular dysplasia/cardiomyopathy-associated desmosomal mutation carriers. J. Am. Coll. Cardiol..

[7] Pilichou K., Thiene G., Bauce B., Rigato I., Lazzarini E., Migliore F., Perazzolo Marra M., Rizzo S., Zorzi A., Daliento L. (2016). Arrhythmogenic cardiomyopathy. Orphanet J. Rare Dis..

[8] Corrado D., Van Tintelen P.J., McKenna W.J., Hauer R.N.W., Anastastakis A., Asimaki A., Basso C., Bauce B., Brunckhorst C., Bucciarelli-Ducci C. (2020). Arrhythmogenic right ventricular cardiomyopathy: Evaluation of the current diagnostic criteria and differential diagnosis. Eur. Heart J..

[9] Corrado D., Perazzolo Marra M., Zorzi A., Beffagna G., Cipriani A., De Lazzari M., Migliore F., Pilichou K., Rampazzo A., Rigato I. (2020). Diagnosis of Arrhythmogenic Cardiomyopathy: The Padua Criteria. Int. J. Cardiol..

[10] Sommariva E., Brambilla S., Carbucicchio C., Gambini E., Meraviglia V., Dello Russo A., Farina F.M., Casella M., Catto V., Pontone G. (2016). Cardiac mesenchymal stromal cells are a source of adipocytes in arrhythmogenic cardiomyopathy. Eur. Heart J..

[11] Uccelli A., Moretta L., Pistoia V. (2008). Mesenchymal stem cells in health and disease. Nat. Rev. Immunol..

[12] Nombela-Arrieta C., Ritz J., Silberstein L.E. (2011). The elusive nature and function of mesenchymal stem cells. Nat. Rev. Mol. Cell Biol..

[13] Bianco P. (2014). “Mesenchymal” stem cells. Annu. Rev. Cell Dev. Biol..

[14] Chelko S.P., Asimaki A., Andersen P., Bedja D., Amat-Alarcon N., DeMazumder D., Jasti R., MacRae C.A., Leber R., Kleber A.G. (2016). Central role for GSK3β in the Pathogenesis of arrhythmogenic cardiomyopathy. JCI Insight.

[15] Pilichou K., Nava A., Basso C., Beffagna G., Bauce B., Lorenzon A., Frigo G., Vettori A., Valente M., Towbin J. (2006). Mutations in desmoglein-2 gene are associated with arrhythmogenic right ventricular cardiomyopathy. Circulation.

[16] Ackers-Johnson M., Li P.Y., Holmes A.P., O’Brien S.M., Pavlovic D., Foo R.S. (2016). A simplified, langendorff-free method for concomitant isolation of viable cardiac myocytes and nonmyocytes from the adult mouse heart. Circ. Res..

[17] Dominici M., Le Blanc K., Mueller I., Slaper-Cortenbach I., Marini F.C., Krause D.S., Deans R.J., Keating A., Prockop D.J., Horwitz E.M. (2006). Minimal criteria for defining multipotent mesenchymal stromal cells. the international society for cellular therapy position statement. Cytotherapy.

[18] Angioni R., Liboni C., Herkenne S., Sánchez-Rodríguez R., Borile G., Marcuzzi E., Calì B., Muraca M., Viola A. (2020). CD73+ extracellular vesicles inhibit angiogenesis through adenosine A2B receptor signalling. J. Extracell. Vesicles.

[19] Zaglia T., Milan G., Ruhs A., Franzoso M., Bertaggia E., Pianca N., Carpi A., Carullo P., Pesce P., Sacerdoti D. (2014). Atrogin-1 deficiency promotes cardiomyopathy and premature death via impaired autophagy. J. Clin. Investig..

[20] Muinao T., Pal M., Boruah H.P.D. (2018). Cytosolic and transmembrane protein extraction methods of breast and ovarian cancer cells: A comparative study. J. Biomol. Tech..

[21] Schindelin J., Arganda-Carreras I., Frise E., Kaynig V., Longair M., Pietzsch T., Preibisch S., Rueden C., Saalfeld S., Schmid B. (2012). Fiji: An open-source platform for biological-image analysis. Nat. Methods.

[22] Tavares S., Vieira A.F., Taubenberger A.V., Araújo M., Martins N.P., Brás-Pereira C., Polónia A., Herbig M., Barreto C., Otto O. (2017). Actin Stress fiber organization promotes cell stiffening and proliferation of pre-invasive breast cancer cells. Nat. Commun..

[23] Giusti B., Margheri F., Rossi L., Lapini I., Magi A., Serratì S., Chillà A., Laurenzana A., Magnelli L., Calorini L. (2013). Correction: Desmoglein-2-integrin beta-8 interaction regulates actin assembly in endothelial cells: Deregulation in Systemic sclerosis. PLoS ONE.

[24] Ebert L.M., Tan L.Y., Johan M.Z., Min K.K.M., Cockshell M.P., Parham K.A., Betterman K.L., Szeto P., Boyle S., Silva L. (2016). A Non-canonical role for Desmoglein-2 in endothelial cells: Implications for neoangiogenesis. Angiogenesis.

[25] Kant S., Freytag B., Herzog A., Reich A., Merkel R., Hoffmann B., Krusche C.A., Leube R.E. (2019). Desmoglein 2 mutation provokes skeletal muscle actin expression and accumulation at intercalated discs in murine hearts. J. Cell Sci..

[26] Hatzfeld M., Haffner C., Schulze K., Vinzens U. (2000). The function of plakophilin 1 in desmosome assembly and actin filament organization. J. Cell Biol..

[27] Bouchet B.P., Akhmanova A. (2017). Microtubules in 3D cell motility. J. Cell Sci..

[28] Yang Y., Qu R., Fan T., Zhu X., Feng Y., Yang Y., Deng T., Peng Y., Huang W., Ouyang J. (2018). Cross-talk between microtubules and the linker of nucleoskeleton complex plays a critical role in the adipogenesis of human adipose-derived stem cells. Stem. Cell Res. Ther..

[29] Biedzinski S., Faivre L., Vianay B., Delord M., Blanchoin L., Larghero J., Théry M., Brunet S. (2019). Microtubules deform the nucleus and force chromatin reorganization during early differentiation of human hematopoietic stem cells. bioRxiv.

[30] Cai W.F., Liu G.S., Wang L., Paul C., Wen Z.L., Wang Y. (2016). Repair injured heart by regulating cardiac regenerative signals. Stem. Cells Int..

[31] El Agha E., Kramann R., Schneider R.K., Li X., Seeger W., Humphreys B.D., Bellusci S. (2017). Mesenchymal stem cells in fibrotic disease. Cell Stem. Cell..

[32] Fischer U.M., Harting M.T., Jimenez F., Monzon-Posadas W.O., Xue H., Savitz S.I., Laine G.A., Cox C.S. (2009). Pulmonary passage is a major obstacle for intravenous stem cell delivery: The pulmonary first-pass effect. Stem. Cells Dev..

[33] Agrimi J., Scalco A., Agafonova J., Williams III L., Pansari N., Keceli G., Jun S., Wang N., Mastorci F., Tichnell C. (2020). Psychosocial stress hastens disease progression and sudden death in mice with arrhythmogenic cardiomyopathy. J. Clin. Med..

[34] Chelko S.P., Asimaki A., Lowenthal J., Bueno-Beti C., Bedja D., Scalco A., Amat-Alarcon N., Andersen P., Judge D.P., Tung L. (2019). Therapeutic modulation of the immune response in arrhythmogenic cardiomyopathy. Circulation.

[35] Hu K.X., Sun Q.Y., Guo M., Ai H.S. (2010). The Radiation Protection and therapy effects of mesenchymal stem cells in mice with acute radiation injury. Br. J. Radiol..

[36] Lopez Perez R., Brauer J., Rühle A., Trinh T., Sisombath S., Wuchter P., Grosu A.L., Debus J., Saffrich R., Huber P.E. (2019). Human mesenchymal stem cells are resistant to UV-B irradiation. Sci. Rep..

