# Supplementary material for: Arrhythmogenic Cardiomyopathy Is a Multicellular Disease Affecting Cardiac and Bone Marrow Mesenchymal Stromal Cells
Source: J Clin Med. 2021 Apr 26;10(9):1871. doi: 10.3390/jcm10091871 (PMC8123444; doi:10.3390/jcm10091871)
Supplement: Supplementary file 1 [file jcm-10-01871-s001.zip › jcm-1184150-supplementary.pdf]

Journal of
Clinical Medicine

Article

Arrhythmogenic Cardiomyopathy is a multicellular disease affecting cardiac and bone marrow mesenchymal stromal cells

Supplementary Content:
- Supplementary Methods
- Supplementary Tables (1–3)
- Supplementary Figures (1–7)
- Supplementary Figure Legends (1–7)

1. Supplementary Methods

SM. 1. Bone marrow processing and staining.

Femurs were harvested from 4-month-old control and *Dsg2*$^{mut/mut}$ mice, cleaned to remove contaminating tissues and muscles, and fixed in 4% paraformaldehyde (PFA, w:v in PBS) at room temperature for 4 hours. After fixation, samples were washed and incubated in 0.5 M EDTA for one week at 4 °C. After 7 days, femurs were embedded in OCT (Kaltek) and frozen in 2-methylbutane (Sigma) cooled by immersion in liquid nitrogen. Ten-micron longitudinal cryosections were obtained with a cryostat Leica CM1950 and used for histological and IF analyses.

SM. 2. Immunofluorescence analysis of femurs sections.

After a brief permeabilization in 1X PBS, supplemented with 1% bovine serum albumin (BSA) and 0.5% Triton X-100 (all from Sigma), femur cryosections were incubated with a rabbit anti-perilipin1 antibody (1:200, Cell Signaling) overnight at 4 °C. The primary antibody was revealed by a 488-conjugated anti-rabbit (1:200, Jackson Lab). Sections were analyzed with a fluorescence microscope Leica DM6B (equipped with the CDD camera DFC300FX and the IM1000 software).

SM. 3. Immunofluorescence analysis of cultured cells.

Cells were fixed in 3.7% formaldehyde (v:v, in PBS) and stained with either AlexaFLUOR®-568 conjugated phalloidin (1:100, Thermo Fisher Scientific) or rabbit anti-α-tubulin antibody (1:100, Sigma). Cells were imaged with a ZEISS LSM900 with Airyscan II confocal microscopy, and images were analyzed to calculate cell area, perimeter, and perimeter/area ratio, using Fiji-ImageJ software [21].

SM. 4. In vitro proliferation assay.

BM- and C-MSCs from both *Dsg2*$^{mut/mut}$ and control mice were cultured in DMEM 1 g/L (Lonza), supplemented with 2mM P/S (Lonza) and 2 mM L-glutamine (Gibco). Cells were incubated for 48 consecutive hours with 5-Bromo-2'-deoxyuridine-BrdU 10 μM BrdU (Sigma) and then processed with the fixation/permeabilization solution kit (BD Cytofix/Cytoperm™), according to manufacturer's instruction. Cells were stained with an AlexaFLUOR®-647 MoBU1-antiBrdU (1:50, Thermo Fisher Scientific) and analyzed with FACS Canto II. Data analysis was performed with FlowJo software.

SM. 5. In vivo proliferation assay.

Two-week-old *Dsg2*$^{mut/mut}$ mice and age- and sex-matched littermate controls were injected with BrdU (100 mg/kg, i.p.; Sigma). After 24 hours, mice were sacrificed, and organs (heart and BM) were processed as described in Materials and Methods. Cells were stained with AlexaFluor647 MoBU1-antiBrdU (1:50, Thermo Fisher Scientific) in

combination with antibodies specific for CD45, CD44, or CD90 (**Suppl. Table 1**). Cells were analyzed with FACS Canto II, and data were analyzed with FlowJo software.

2. Supplementary Tables

Supplementary Table S1. List of antibodies used in this study for FACS analyses.

Antibody	Supplier
PerCP anti-CD45	BD
PE anti-CD117	Miltenyi Biotech
Pacific Blue™ anti-mouse CD73	BioLegend
APC anti-CD11b	BD
I-A/I-E MHCI FITC	BD
PE anti-mouse Ly-6A/E (Sca-1)	BioLegend
PE anti-CD90	BD
APC anti-CD44	BioLegend
FITC anti-mouse CD106	BioLegend

Copyright: © 2021 by the authors. Licensee MDPI, Basel, Switzerland. This article is an open access article distributed under the terms and conditions of the Creative Commons Attribution (CC BY) license (http://creativecommons.org/licenses/by/4.0/).

Supplementary Table S2. Oligos used in this study for RTqPCR analysis.

gene	Forward primer	Reverse primer
Dsc2	5′-CTGTGGGATCTATGCGCTCC-3′	5′-TCCATCAGGTTCACTCTGCC-3′
Dsg2 C-term	5′-GTGCGCCATTCAGCTTCTCC-3′	5′-AGTGAGCTGAAGGACCTGCC-3′
Dsg2 N-term	5′-TGCTTGGACTTTGGAAACGGAC-3′	5′-TTCTGGACAGGTCTTCGCCC-3′
Dsg2 tot	5′-ACGCACCAGGAAAGTACCAG-3′	5′-GAGCTGAAGGACCTGCCTTT-3′
Dsp	5′-CAAAAGCAGGCTTTAGAGGCAT-3′	5′-CTTCACCAGCAGGCTCTCTC-3′
Gapdh	5′-CACCATCTTCCAGGAGCGAG-3′	5′-CCTTCTCCATGGTGGTGAAGAC-3′
Jup	5′-ATCCACGCCATCCTGAGAGC-3′	5′-CACAGAGCCAGGTTCCGGAT-3′
Pkp2	5′-CCACAGCCTCTGCTTGCTAT-3′	5′-CTTGTTGGGGGCATAGCCTT-3′

Supplementary Table S3. List of primary antibodies used in this study for IF and WB analyses. m, mouse; Rb, rabbit.

Antibody	Dilution	Supplier
DSC2 (m)	1:200	Santa Cruz
DSG2 C-term (Rb)	1:1000	Abcam
GAPDH (m)	1:10.000	Abcam
JUP (Rb)	1:500	Abcam
Perilipin-1 (Rb)	1:200	Cell Signaling
Phalloidin AlexaFLUOR®-568	1:100	Thermo Fisher Scientific
PKP2 (m)	1:100	Progen
α-Tubulin (m)	1:100	Sigma

3. Supplementary Figures

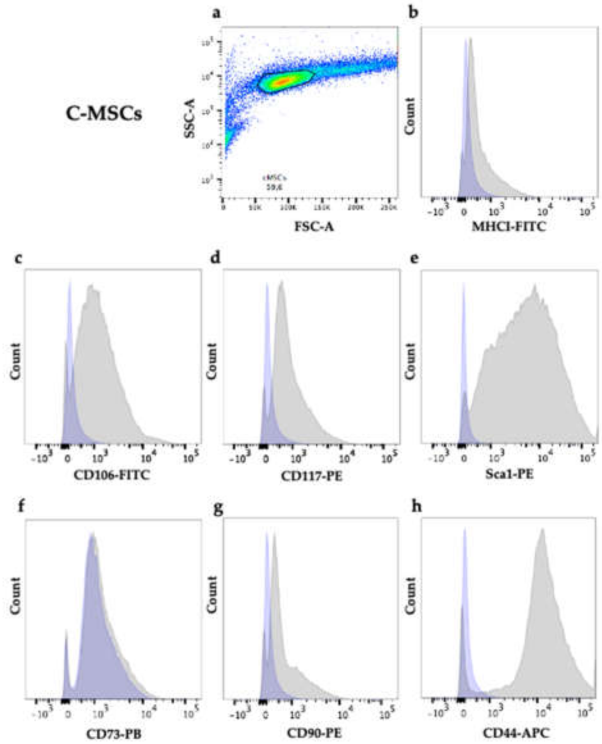

Figure S1. Flow cytometry characterization of cardiac mesenchymal stromal cells (C-MSCs)**.** (**a**) Representative dot-plot of cultured control C-MSCs gated according to forward scatter (FSC-A) and side scatter (SSC-A). (**b–h**) Cells were stained with a panel of different antibodies to define the mesenchymal stromal cell phenotype. Histograms show the expression of each marker (indicated on the *x* axis of the respective histogram) within the $CD45^-/CD11b^-$ subset (grey area). The blue area refers to unstained cells, used as negative control.

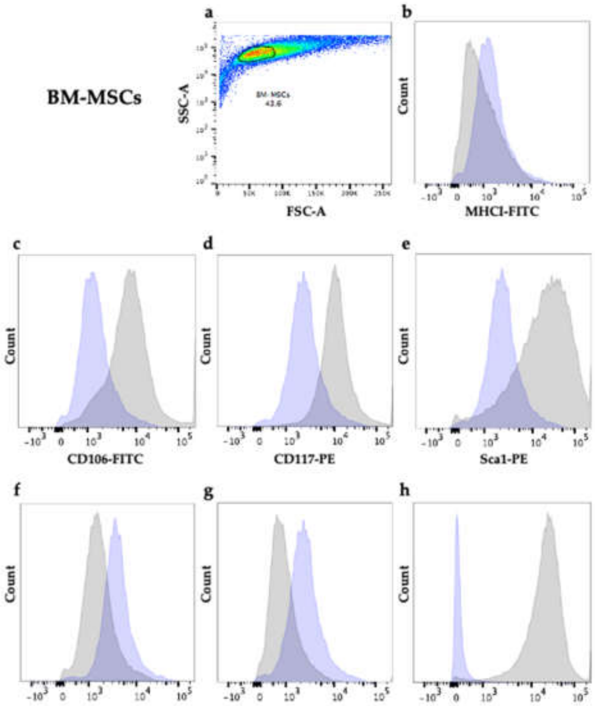

Figure S2. Flow cytometry characterization bone marrow-mesenchymal stromal cells (BM-MSCs). (**a**) Representative dot-plot of cultured control BM-MSCs gated according to forward scatter (FSC-A) and side scatter (SSC-A). (**b–h**) Cells were stained with a panel of different antibodies to define the mesenchymal stromal cell phenotype. Histograms show the expression of each marker (indicated in the *x* axis of the respective histogram) within the $CD45^{-}/CD11b^{-}$ subset (grey area). The blue area refers to unstained cells, used as negative control.

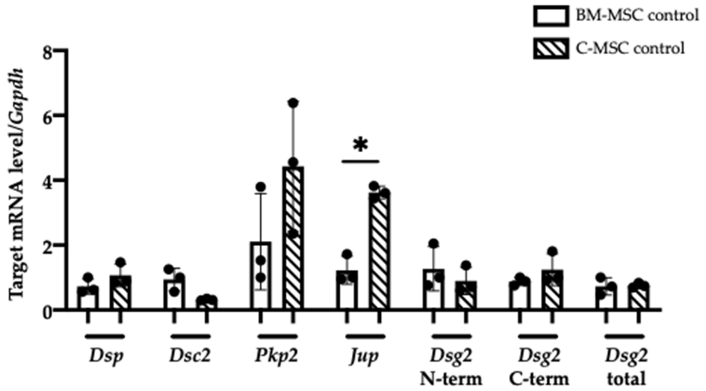

Figure S3. Cardiac- and bone barrow-mesenchymal stromal cells (MSCs) express desmosomal genes. RT-qPCR on extracts from cultured cardiac- and BM-derived MSCs isolated from adult control mice to assess the expression levels of desmosomal genes. Bars represent mean, and error bars represent the relative standard deviation (SD). $n = 3$ samples for each group. The experiment was repeated three times. * $p \leq 0.05$. *Dsp*, desmoplakin; *Dsc*2, desmocollin-2; *Dsg2*, desmoglein-2; *Jup*, plakoglobin; *Pkp2*, plakophilin-2.

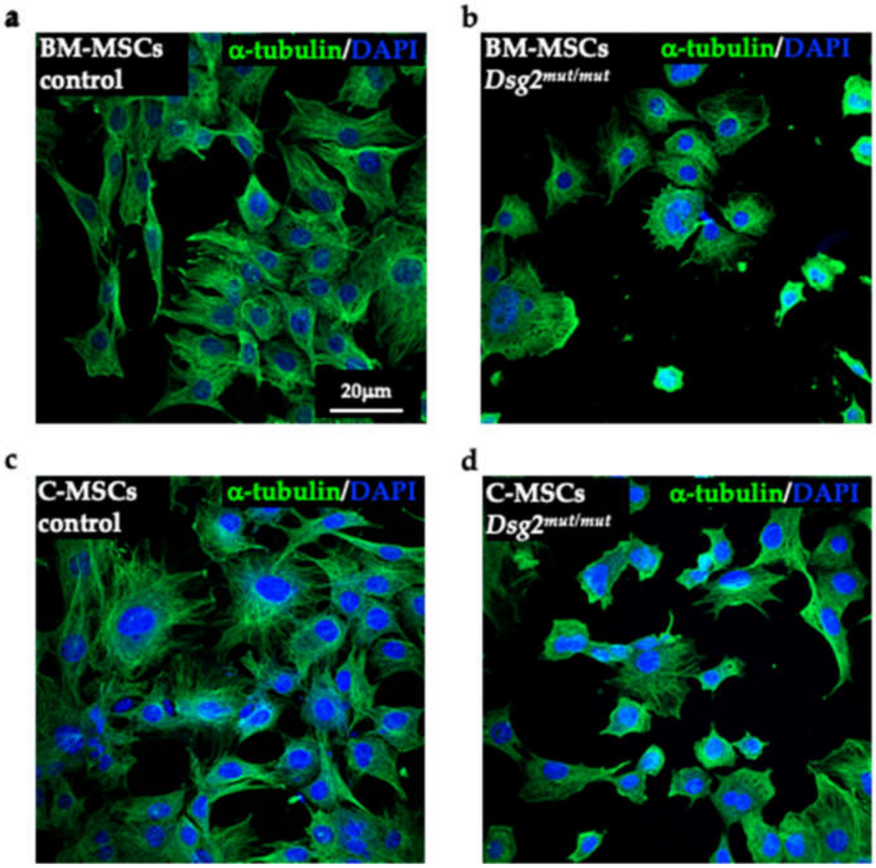

Figure 4. AC-linked *Dsg2* variant affects microtubule organization in both cardiac- and bone marrow-mesenchymal stromal cells. (**a**–**d**) Confocal IF analysis of cultured BM- (**a**–**b**) and cardiac- (**c**–**d**) MSCs, stained with an antibody specific for α-tubulin (green signal). Nuclei were counterstained with DAPI (blue signal).

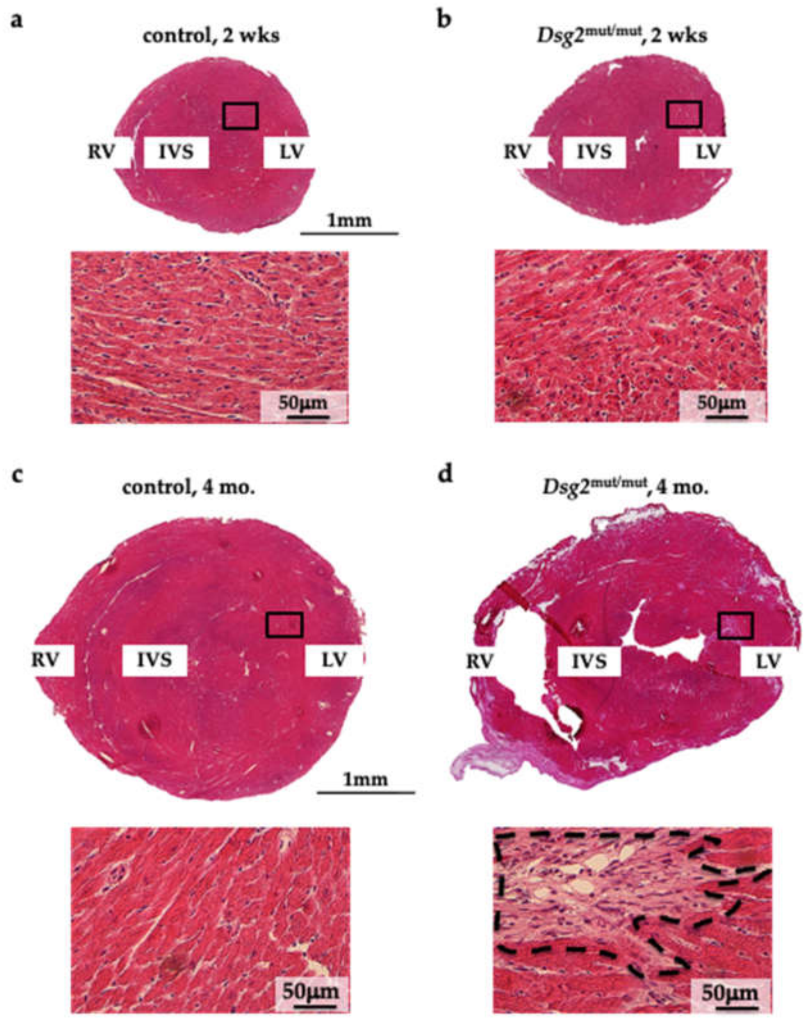

Figure 5. AC-linked *Dsg2* variant progressively affects heart morphology. (**a**–**d**) Hematoxylin-eosin staining of heart cryosections, from the mid portion of the ventricles, from 2-week-old (**a**–**b**) vs. 4-month-old (**c**–**d**) control (**a**, **c**) and *Dsg2*$^{mut/mut}$ (**b**, **d**) mice. Bottom images in (**a**–**d**) are high magnifications of the black boxes in the corresponding heart sections. RV, right ventricle; IVS, interventricular septum; LV, left ventricle.

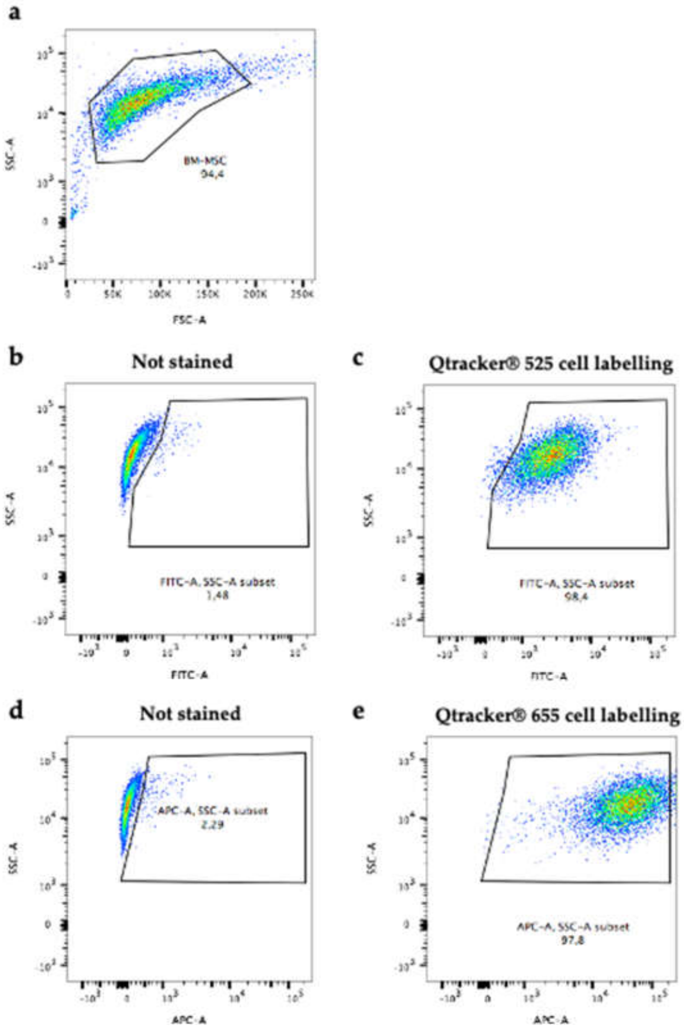

Figure 6. Flow cytometry characterization of the efficiency of BM-MSC labelling with Qtracker®. (**a**) Representative dot-plot of BM-MSCs gated according to forward scatter (FSC-A) and side scatter (SSC-A). (**b–e**) Representative dot-plots of unstained BM-MSCs (**b**,**d**) vs. cells labelled with Qtracker® 525 (**c**) or Qtracker® 655 (**d**). (**b**) and (**d**) are the controls of (**c**) and (**e**), respectively.

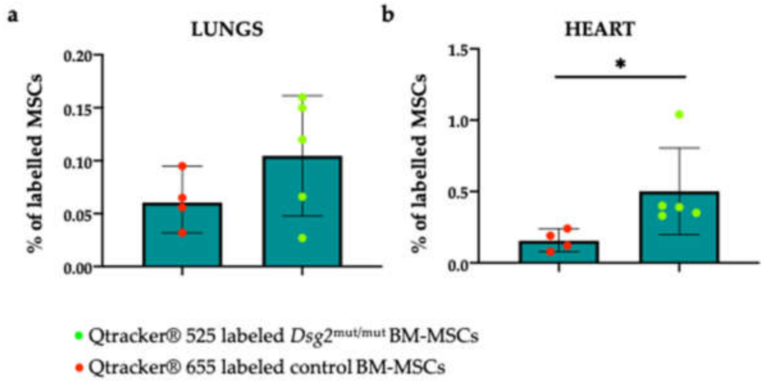

Figure 7. *Dsg2*$^{mut/mut}$ bone marrow-mesenchymal stromal cells preferentially home to the heart, compared with control BM-MSCs. (**a–b**) Ex vivo flow cytometry analysis of lungs (**a**) and hearts (**b**) of adult *Dsg2*$^{mut/mut}$ mice injected with either AC or control BM-MSCs, labelled with Qtracker® 525- (in green) or Qtracker® 655- (in red), respectively. The percentage of labelled cells per organ

normalized over the total number of cells obtained from enzymatic digestion was evaluated. Bars represent the median with 95% confidence interval (CI). $n = 4$ mice for each group. * $p \leq 0.05$.
